# Impact of Demographic and Clinical Subgroups in Google Trends Data: Infodemiology Case Study on Asthma Hospitalizations

**DOI:** 10.2196/51804

**Published:** 2025-03-10

**Authors:** Diana Portela, Alberto Freitas, Elísio Costa, Mattia Giovannini, Jean Bousquet, João Almeida Fonseca, Bernardo Sousa-Pinto

**Affiliations:** 1 Department of Community Medicine, Information and Health Decision Sciences Faculty of Medicine University of Porto Porto Portugal; 2 Center for Health Technologies and Services Research Health Research Network University of Porto Porto Portugal; 3 Research Unit on Applied Molecular Biosciences Faculty of Pharmacy University of Porto Porto Portugal; 4 Allergy Unit, Department of Pediatrics Meyer Children’s Hospital Istituto di Ricovero e Cura a Carattere Scientifico Florence Italy; 5 Department of Health Sciences University of Florence Florence Italy; 6 Institute of Allergology Charité – Universitätsmedizin Berlin Corporate Member of Freie Universität Berlin and Humboldt-Universität zu Berlin Berlin Germany; 7 Fraunhofer Institute for Translational Medicine and Pharmacology ITMP, Allergology and Immunology Berlin Germany; 8 MASK-air Montpellier France

**Keywords:** infodemiology, asthma, administrative databases, multimorbidity, co-morbidity, respiratory, pulmonary, Google Trends, correlation, hospitalization, admissions, autoregressive, information seeking, searching, searches, forecasting

## Abstract

**Background:**

Google Trends (GT) data have shown promising results as a complementary tool to classical surveillance approaches. However, GT data are not necessarily provided by a representative sample of patients and may be skewed toward demographic and clinical groups that are more likely to use the internet to search for their health.

**Objective:**

In this study, we aimed to assess whether GT-based models perform differently in distinct population subgroups. To assess that, we analyzed a case study on asthma hospitalizations.

**Methods:**

We analyzed all hospitalizations with a main diagnosis of asthma occurring in 3 different countries (Portugal, Spain, and Brazil) for a period of approximately 5 years (January 1, 2012-December 17, 2016). Data on web-based searches on common cold for the same countries and time period were retrieved from GT. We estimated the correlation between GT data and the weekly occurrence of asthma hospitalizations (considering separate asthma admissions data according to patients’ age, sex, ethnicity, and presence of comorbidities). In addition, we built autoregressive models to forecast the weekly number of asthma hospitalizations (for the different aforementioned subgroups) for a period of 1 year (June 2015-June 2016) based on admissions and GT data from the 3 previous years.

**Results:**

Overall, correlation coefficients between GT on the pseudo-influenza syndrome topic and asthma hospitalizations ranged between 0.33 (in Portugal for admissions with at least one Charlson comorbidity group) and 0.86 (for admissions in women and in White people in Brazil). In the 3 assessed countries, forecasted hospitalizations for 2015-2016 correlated more strongly with observed admissions of older versus younger individuals (Portugal: Spearman ρ=0.70 vs ρ=0.56; Spain: ρ=0.88 vs ρ=0.76; Brazil: ρ=0.83 vs ρ=0.82). In Portugal and Spain, forecasted hospitalizations had a stronger correlation with admissions occurring for women than men (Portugal: ρ=0.75 vs ρ=0.52; Spain: ρ=0.83 vs ρ=0.51). In Brazil, stronger correlations were observed for admissions of White than of Black or Brown individuals (ρ=0.92 vs ρ=0.87). In Portugal, stronger correlations were observed for admissions of individuals without any comorbidity compared with admissions of individuals with comorbidities (ρ=0.68 vs ρ=0.66).

**Conclusions:**

We observed that the models based on GT data may perform differently in demographic and clinical subgroups of participants, possibly reflecting differences in the composition of internet users’ health-seeking behaviors.

## Introduction

The assessment of internet users’ behavior can be a valuable source of information regarding their specific interests, preferences, and perceptions pertaining to diverse health topics. Such an assessment not only enables the identification and exploration of emerging trends in health-related interests but also facilitates an understanding of the factors influencing health information seeking, dissemination, and consumption in the digital age [1,2]. In this context, there are different methodological approaches that can be used, including the assessment of the relative volume of searches on specific health topics and keywords (i.e, assessing what internet users seek) [3-7] or the assessment of content available online, including social media posts by internet users [8-10]. These approaches are part of a recent field of studies termed “infodemiology,” which is defined as “the science of distribution and determinants of information in an electronic medium, specifically the Internet, or in a population, with the ultimate aim to inform public health and public policy” [1,11].

Infodemiology studies have been conducted to accomplish different goals [7,12,13]. For instance, Google Trends (GT) data, which measure the relative volume of searches on a specific topic or term, have shown promising results as a complementary tool to classical surveillance methods [6], in forecasting influenza spread and hospitalizations [14-16], for modelling COVID-19 spread [17,18], and for forecasting asthma admissions [19]. However, GT data are not necessarily provided by a representative sample of individuals within a certain country or region [20] but can rather preferentially reflect demographic or clinical groups that are more likely to use the internet for health-related inquiries. For instance, the online behavior of younger, more educated, or technologically literate individuals may be overrepresented in GT data. Moreover, health-related search behaviors can be influenced by a host of factors, including the severity and type of health conditions, the availability and quality of health information online, and individual health literacy levels. Therefore, it is possible to hypothesize that GT data should not be seen as a “one-size-fits-all” tool for health research since we do not know the clinical and demographic composition of the individuals searching for a specific health term or topic. As such, it is probable that there may be relevant differences from what is observed in the general population, with relevant implications for the performance and interpretability of GT-based models.

Therefore, in this study, we aimed to assess whether GT-based models can have a different performance when considering different population subgroups (according to their clinical and demographic characteristics). To achieve that goal, we assessed a case study of asthma hospitalizations. Specifically, we (1) assessed the correlation between GT data for the *common cold* and the number of hospitalizations for asthma considering admissions of subgroups of patients (according to their age, sex, ethnicity, and presence of comorbidities) and (2) compared the performance of models predicting asthma hospitalizations based on GT for these specific participant segments (according to their age, sex, ethnicity, and presence of comorbidities).

## Methods

### Study Design

This study adhered to the methodological framework proposed by Mavragani and Ochoa [21]. In a previous study by our team, we had (1) established a correlation between GT data related to common cold–related search terms and asthma hospitalizations and (2) evaluated whether GT data on the *common cold*, combined with data on admissions, could help forecast asthma hospitalizations. In this study, we applied the same methodology (correlations and forecast models) and used the same GT data but specifically considered those hospital admissions occurring in patients of each sex, age group (18-64 years old versus ≥65 years old), ethnicity (White versus Black or Brown [“pardo”]), and the presence or absence of at least one Charlson comorbidity [22]. We assessed a period of approximately 5 years (2012-2016), assessing data from Portugal, Spain, and Brazil.

### Data Sources and Variables

#### GT Data

GT is a tool that offers insight into the popularity of search terms by providing their relative search volume data on a scale of 0 to 100 (where 100 represents the peak interest at a specific time and location). It allows users to compare the popularity of different keywords, topics, or queries across regions and time periods. The data are indexed to show the proportion of searches for a specific term relative to all searches on Google at that specific time and location [6,12,23].

We obtained GT data on January 13, 2020, as already described by Sousa-Pinto et al [19]. In brief, we retrieved country-level GT on rhinovirus-related search terms for 5 years (between 2012 and 2016) in Portugal, Spain, and Brazil: 5 years is the maximum amount of time for which GT displays data on a weekly level. These countries and timeframe were chosen (1) to allow comparability with a previous study [19] and (2) due to the accessibility of nationwide data regarding the frequency of weekly hospitalizations presented by age and sex. No specific categories or subcategories of GT data were selected. We accessed GT data exclusively through its web interface, with a single data extraction performed for each country included in the study.

For each country, we applied 2 different GT queries. The first query focused on the *pseudo-influenza syndrome topic*, which was subsequently renamed as the *common cold* topic (of note, “topics” encompass groups of search terms associated with a specific concept regardless of language [12,19,21,24]). The second query consisted of a combination of search terms related to the *common cold*, carefully selected through discussions with native speakers of each language:

- Portugal: constipação + resfriado
- Spain: resfriado + resfrío + catarro + constipado + refredat + constipate + arrefriado + hotzeri
- Brazil: resfriado

We did not include quotation marks for the search terms as each term represented a single word. Misspellings or nonaccentuated forms were also excluded from the search term combinations, as we identified identical relative search volumes observed whenever misspelt words were or were not included in search term combinations.

#### Asthma Hospitalization Data Sources

We analyzed hospitalization data from January 1, 2012, to December 17, 2016 (we excluded the last 2 weeks of 2016 due to unavailable information on discharges in Portugal and Brazil—as many patients admitted toward the end of 2016 were discharged in 2017). In the 3 countries under investigation, we examined all hospitalizations in which asthma was identified as the primary diagnosis. Specifically, we used the International Classification of Diseases, Ninth Revision, Clinical Modification (ICD-9-CM), code 493.x or International Classification of Diseases, Tenth Revision (ICD-10-CM) [25], code J45.x to identify these cases. The hospitalization data were obtained from the following sources: (1) the Hospital Morbidity database, provided by the Portuguese Central Administration of the Healthcare System, for Portugal [26]; (2) the Hospital Morbidity Survey databases (*Encuesta de morbilidad hospitalaria, Instituto Nacional de Estadistica*) for Spain [27]; and (3) Departamento de Informática do Sistema Único de Saúde (DATASUS) data from the Single Health System (*Sistema Único de Saúde*) for Brazil [28].

For each country, separate analyses were performed by participants’ sex and age group (we considered only the age groups 18-64 years and ≥65 years to ensure a sufficient weekly number of hospital admissions for each analysis). Based on data availability for Portugal [20], analyses were also separately performed for episodes with at least one comorbidity from the Charlson comorbidity group and without any comorbidity from the Charlson comorbidity group. Likewise, based on data availability for Brazil, analyses were also separately performed by participants’ ethnicity (White versus Black or Brown). Information on ethnicity was not available for Portugal and Spain, and information on the presence of comorbidities was not available for Spain and Brazil.

### Statistical Analysis

Data analysis encompassed 2 different steps: (1) assessing the correlations between GT data and asthma hospitalizations in each country after applying time series analysis methods and (2) building models forecasting asthma hospitalizations for a period of 1 year based on GT and hospitalization data from the previous 4 years. To evaluate the predictive capability of the models, we compared the forecasted asthma admissions with the observed hospitalization data. For both GT and the frequency of hospitalizations, we worked with data displayed on a weekly basis (as it allowed detection of short-term variations while mitigating the impact of large random fluctuations that can occur when data are examined on a daily basis).

First, we performed a cross-correlation analysis to examine the correlation between GT data and asthma hospitalizations (cross-correlation can be understood as a statistical method used to analyze the relationship [correlate] between 2 continuous variables that can be measured or sampled at different points in time) [29]. Given that (1) for GT data, a relevant secular trend was expected (reflecting an increase in Google searches over time) and (2) GT and hospitalization data are expressed on different scales (GT results are expressed as relative search values [ie, percentages in relation to the maximum observed value of the whole period], whereas hospitalizations are expressed as absolute values), we removed the secular trend component for both GT and hospitalization data then assessed the correlation between search volumes and asthma hospitalizations. We analyzed Spearman correlations between GT and hospitalization data registered in the same week, as well as cross-correlation coefficients for a lag of 1 and 2 weeks to determine if search volumes demonstrated a stronger correlation with asthma hospitalizations occurring afterward rather than those happening concurrently. Correlation coefficients were presented alongside 95% CIs, which were computed using bootstrap methods for Spearman correlation coefficients.

Second, we built seasonal autoregressive integrated moving average (ARIMA) models to forecast variations in asthma hospitalizations over a period of 1 year [19,30]. Seasonal ARIMA models are used to forecast time series data that exhibit repeating patterns over fixed intervals (in this case, yearly cycles). These models take into account both the nonseasonal patterns and the seasonal variations in the data to make accurate predictions for future time points [31]. For each analysis, seasonal ARIMA models parameters *(p, d, q)(P', D, Q)s* were defined, where *p* denotes the order of autoregression, *d* denotes the degree of difference, *q* denotes the order of the moving average part, *P'* denotes the seasonal order of autoregression, *D* denotes the degree of difference following seasonal integration, *Q* denotes the seasonal moving average, and *s* denotes the length of the seasonal period. We chose *d* and *D* so that the 2012-2016 time series appeared stationary (ie, with constant variance and no extreme fluctuations or overall increasing or decreasing behavior), including by testing a measure of seasonal strength [32]; we chose *s*=52 weeks (since there are roughly 52 weeks in a year); we chose *p* and *P'* based on spikes in partial autocorrelation function plots; and we chose *q* and *Q* based on spikes in autocorrelation function plots. Identification of these parameters using autocorrelation and partial autocorrelation plots allowed us to define candidate models. Final seasonal ARIMA models were then selected based on the results of the Ljung-Box test (which was applied to assess whether residuals look like white noise) and on the minimization of the Akaike information criteria and Bayesian information criteria (see Table S1 in Multimedia Appendix 1 for the parameters defined for each model). In this study, we used asthma hospitalization data alongside GT data to forecast future asthma hospitalizations. The data set was split into a training set and a testing set. Specifically, the training set comprised asthma hospitalizations and GT data collected from July 1, 2012, to June 20, 2015. We then used this trained model to forecast asthma hospitalizations for the testing set, which included hospitalizations between the weeks of June 21, 2015, and June 19, 2016. This split allowed for the evaluation of model performance on previously unseen data.

To evaluate the predictive performance of the models, several measures were used: (1) the Spearman correlation coefficients between the predicted variation in hospitalizations and the actual number of asthma hospitalizations (ie, without time series decomposition), (2) the average weekly difference between the numbers of predicted and observed hospitalizations, and (3) the number of weeks whose number of observed asthma hospitalizations fell outside the 95% CI for predicted admissions.

All analyses were performed using R software, version 4.3.0 (R Foundation for Statistical Computing) [33], using the *forecast* and *urca* packages.

### Ethical Considerations

#### Ethics Approval

The data used in this study were provided by the Central Administration of the Health System (Administração Central do Sistema de Saúde [ACSS]) in accordance with their institutional data-sharing policies. These data consist of the Morbidity Hospital Database (Bases de Dados de Morbilidade Hospitalar), which includes anonymized and de-identified data. Per the ACSS’s internal guidelines, data anonymization and de-identification are conducted before any access is granted to external researchers. As a result, specific ethical approval was not required, as the use of anonymized data aligns with both Portuguese data protection regulations and the institutional policy governing secondary data analysis. [34-36],

#### Privacy and Confidentiality

The ACSS guarantees that the provided data sets are fully anonymized, making it impossible to identify individual patients. In addition, strict data use agreements are in place, which ensure that external entities, such as the authors of this study, commit to (1) using the data exclusively for research within the scope of their project, ensuring secure and fair data processing; (2) requesting explicit authorization from ACSS for any other use beyond the agreed scope; (3) not sharing the data with third parties; (4) citing ACSS as the source of the data in any resulting publications; (5) providing ACSS with copies of all publications that use the data; and (6) taking full responsibility for any analysis or conclusions drawn from the provided data sets.

#### Compensation

No compensation was provided, as the study did not involve direct patient recruitment or interaction.

Additionally, any identification of specific hospitals or the disclosure of medical device pricing data requires explicit approval from the respective institutions. This confidentiality further strengthens the protection of sensitive information while allowing for the comprehensive analysis of anonymized data.

## Results

**Figure 1:**
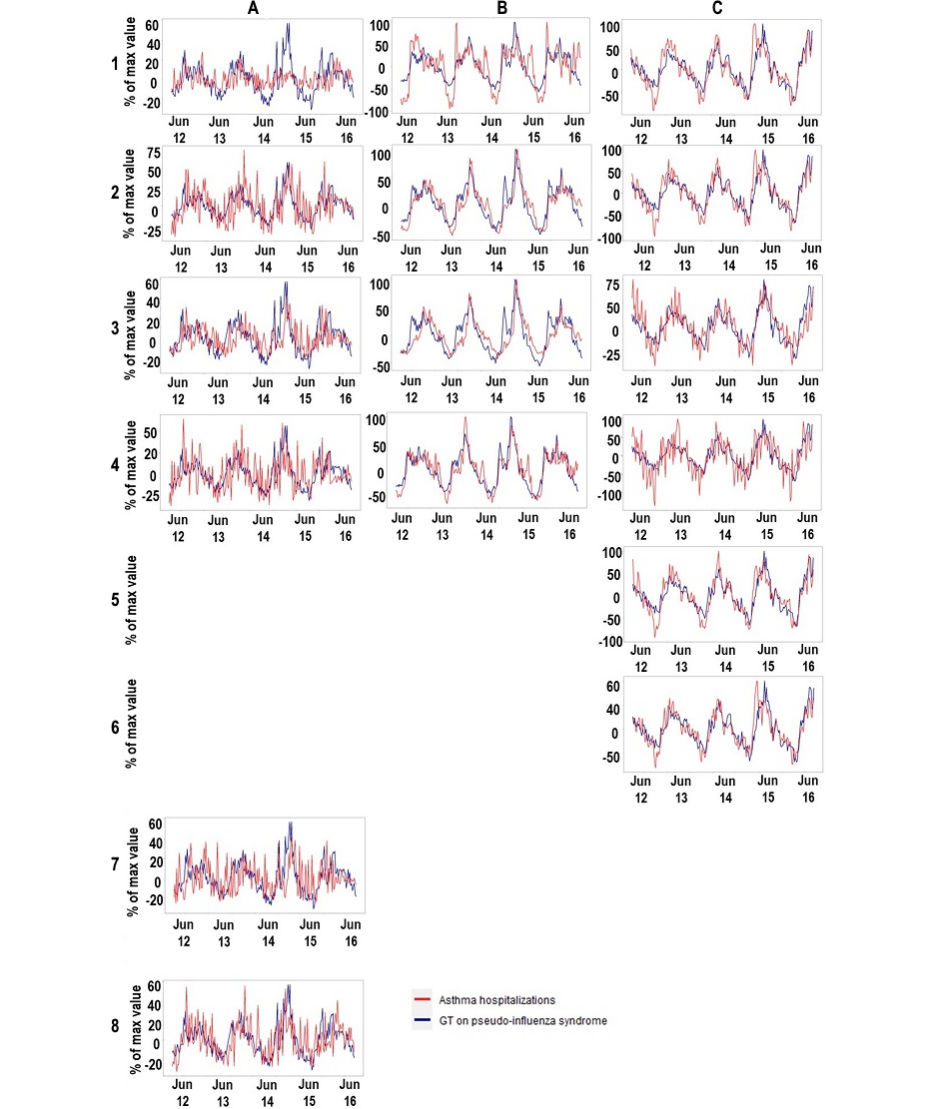
Google Trends (GT) data on pseudo-influenza syndrome and asthma hospitalizations (2012-2016) for (A.1) men in Portugal, (B.1) men in Spain, (C.1) men in Brazil, (A.2) women in Portugal, (B.2) women in Spain, (C.2) women in Brazil, (A.3) older adults in Portugal, (B.3) older adults in Spain, (C.3) older adults in Brazil, (A.4) younger adults in Portugal, (B.4) younger adults in Spain, (C.4) younger adults in Brazil, (C.5) White people in Brazil, (C.6) Black or Brown people in Brazil, (A.7) people with comorbidities in Portugal, and (A.8) people without comorbidities in Portugal. The trend component of the time series has been plotted after removing the seasonal effects and random error components.

Between 2012 and 2016, GT data for *pseudo-influenza syndrome* presented similar patterns across the 3 countries for which GT data were plotted, with peaks in the winter and valleys in the summer of the respective hemispheres (Figure 1). This pattern was also observed for asthma hospitalizations in each subgroup of patients in each country.

In the assessed countries and for each subgroup of admissions, correlations between GT on the *pseudo-influenza syndrome* topic (after removing the trend component) and asthma hospitalizations ranged between 0.33 (in Portugal for admissions with at least one Charlson comorbidity group) and 0.86 (for admissions of women and White individuals in Brazil; Table 1). Similar values were observed when analyzing the correlations between GT and terms for the *common cold*. In the 3 countries, stronger correlation coefficients were observed for admissions occurring for women. In Portugal and Spain, stronger correlations were found for admissions of younger individuals, while in Brazil, the inverse phenomenon occurred. In Brazil, no differences were observed between correlations for admissions of patients of White or Black/Brown ethnicity. In Portugal, stronger correlations were observed for admissions of patients without comorbidities than for those with at least one comorbidity.

In most cases, GT on the *pseudo-influenza syndrome* topic correlated more strongly with asthma hospitalizations occurring in the subsequent week than with those occurring in the same week (Table 1).

**Figure 2:**
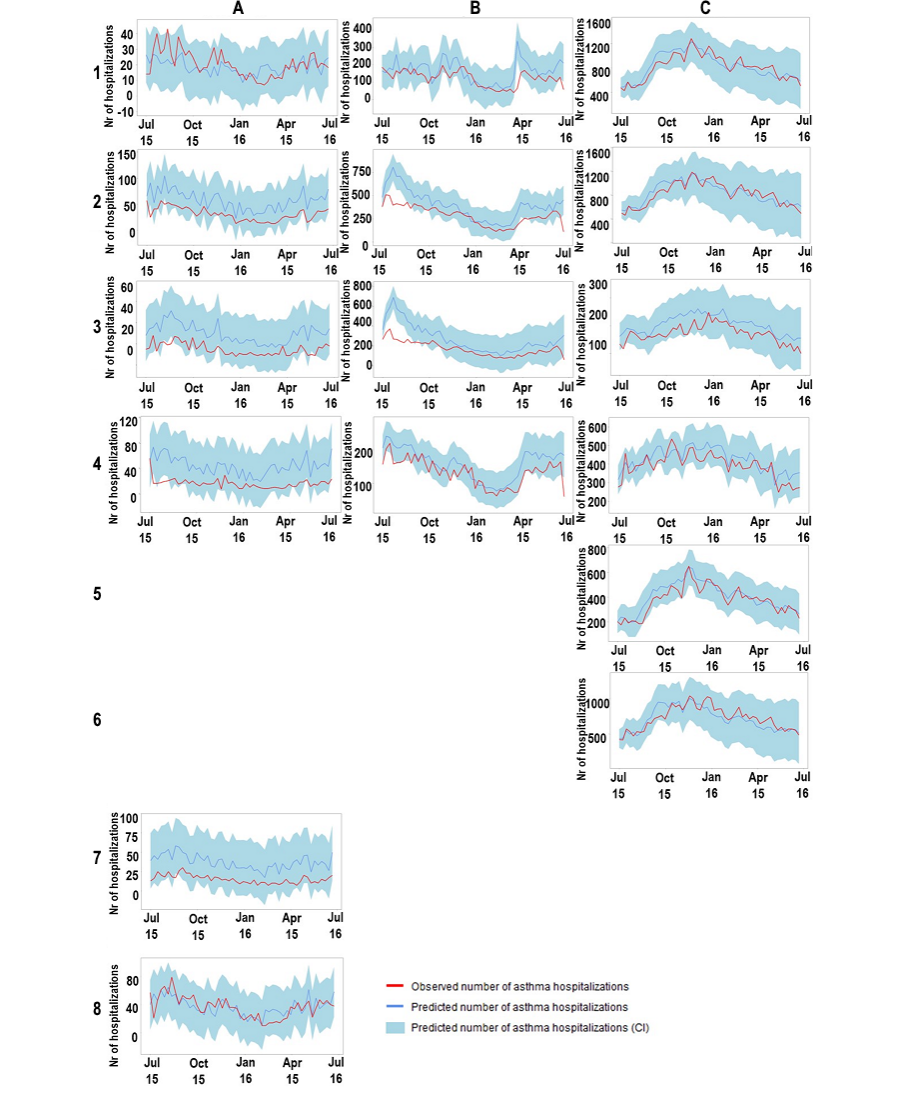
Predicted number (estimated based on previous hospitalizations and Google Trends data) and observed number of asthma hospitalizations (June 2015 to June 2016) for (A) men in Portugal, (B) men in Spain, (C) men in Brazil, (D) women in Portugal, (E) women in Spain, (F) women in Brazil, (G) older adults in Portugal, (H) older adults in Spain, (I) older adults in Brazil, (J) younger adults in Portugal, (K) younger adults in Spain, (L) younger adults in Brazil, (M) White people in Brazil, (N) Black or Brown people in Brazil, (O) people with comorbidities in Portugal, and (P) people without comorbidities in Portugal.

In the 3 assessed countries (Table 2; Figure 2), forecasted hospitalizations for 2015-2016 obtained through seasonal ARIMA models correlated more strongly with observed admissions of older adults versus younger individuals (Portugal: correlation coefficient [ρ]= 0.70 vs ρ= 0.56; Spain: ρ=0.88 vs ρ=0.76; Brazil: ρ=0.83 vs ρ=0.82). In Portugal and Spain, forecasted hospitalizations displayed a much stronger correlation with admissions occurring for women than for men (Portugal: ρ=0.75 vs ρ=0.52; Spain: ρ=0.83 vs ρ=0.51). Consistent results were observed when performing a sensitivity analysis by age group (Table S2 in Multimedia Appendix 1). In Brazil, stronger correlations were observed for admissions of White individuals than of Black or Brown individuals (ρ=0.92 vs ρ=0.87). In Portugal, stronger correlations were observed for admissions of individuals without any comorbidity compared with admissions of individuals with comorbidities (ρ=0.68 vs ρ=0.66). The numbers of weeks with observed hospitalizations outside the confidence interval for predicted values ranged between 1 and 7 for Portugal, 2 and 12 for Spain, and 0 and 1 for Brazil (according to subgroups of admissions in each country).

**Table 1:** Correlation and cross-correlation coefficients between common cold Google Trends data (ie, Google Trends data on the pseudo-influenza syndrome topic and on common cold search terms) and asthma hospitalizations (according to age, sex, ethnicity, and presence of comorbidities) for the period 2012-2016.

Categories					Results based on observed data, correlation coefficients (95% CI)		Results after removal of the trend component, cross-correlation coefficients (95% CI)				
							Week lag –0		Week lag –1		Week lag –2
**Pseudo-influenza syndrome topic**											
	**Portugal**										
		**Sex**									
			Male	0.41 (0.30 to 0.52)		0.36 (–0.17 to 0.17)		0.37 (–0.17 to 0.17)		0.36 (–0.16 to 0.16)	
			Female	0.47 (0.36 to 0.58)		0.44 (–0.19 to 0.19)		0.51 (–0.20 to 0.20)		0.47 (–0.19 to 0.19)	
		**Age group (years)**									
			>65	0.38 (0.25 to 0.50)		0.37 (–0.22 to 0.22)		0.47 (–0.22 to 0.22)		0.49 (–0.21 to 0.21)	
			18-64	0.43 (0.31 to 0.54)		0.41 (–0.15 to 0.15)		0.42 (–0.16 to 0.16)		0.34 (–0.16 to 0.16)	
		**Comorbidities**									
			With comorbidities	0.33 (0.19 to 0.46)		0.32 (–0.18 to 0.18)		0.38 (–0.18 to 0.18)		0.38 (–0.18 to 0.18)	
			Without comorbidities	0.51 (0.40 to 0.61)		0.36 (–0.17 to 0.17)		0.37 (–0.16 to 0.16)		0.36 (–0.17 to 0.17)	
	**Spain**										
		**Sex**									
			Male	0.63 (0.53 to 0.72)		0.65 (–0.34 to 0.34)		0.61 (–0.35 to 0.35)		0.57 (–0.35 to 0.35)	
			Female	0.79 (0.72 to 0.84)		0.83 (–0.39 to 0.39)		0.86 (–0.40 to 0.40)		0.86 (–0.40 to 0.40)	
		**Age group (years)**									
			>65	0.69 (0.60 to 0.76)		0.73 (–0.41 to 0.41)		0.80 (–0.40 to 0.40)		0.82 (–0.40 to 0.40)	
			18-64	0.81 (0.75 to 0.86)		0.83 (–0.34 to 0.34)		0.86 (–0.34 to 0.34)		0.83 (–0.34 to 0.34)	
	**Brazil**										
		**Sex**									
			Male	0.85 (0.80 to 0.88)		0.82 (–0.37 to 0.37)		0.75 (–0.37 to 0.37)		0.68 (–0.37 to 0.37)	
			Female	0.86 (0.82 to 0.89)		0.83 (–0.34 to 0.34)		0.78 (–0.33 to 0.33)		0.72 (–0.33 to 0.33)	
		**Age group (years)**									
			>65	0.74 (0.67 to 0.78)		0.68 (–0.29 to 0.29)		0.70 (–0.29 to 0.29)		0.69 (–0.30 to 0.230)	
			18-64	0.70 (0.63 to 0.75)		0.65 (–0.27 to 0.27)		0.63 (–0.27 to 0.27)		0.59 (–0.27 to 0.27)	
		**Ethnicity**									
			White	0.86 (0.82 to 0.89)		0.84 (–0.35 to 0.35)		0.79 (–0.35 to 0.35)		0.73 (–0.35 to 0.35)	
			Black or Brown	0.85 (0.80 to 0.88)		0.81 (–0.32 to 0.32)		0.75 (–0.32 to 0.32)		0.69 (–0.32 to 0.32)	
**Common cold search terms**											
	**Portugal**										
		**Sex**									
			Male	0.35 (0.23 to 0.48)		0.31 (–0.16 to 0.16)		0.30 (–0.16 to 0.16)		0.34 (–0.16 to 0.16)	
			Female	0.45 (0.33 to 0.56)		0.46 (–0.19 to 0.19)		0.46 (–0.18 to 0.18)		0.52 (–0.19 to 0.19)	
		**Age group (years)**									
			>65	0.37 (0.24 to 0.50)		0.41 (–0.20 to 0.20)		0.45 (–0.20 to 0.20)		0.49 (–0.20 to 0.20)	
			18-64	0.42 (0.30 to 0.53)		0.41 (–0.15 to 0.15)		0.38 (–0.15 to 0.15)		0.42 (–0.16 to 0.16)	
		**Comorbidities**									
			With comorbidities	0.33 (0.19 to 0.45)		0.34 (–0.16 to 0.16)		0.35 (–0.16 to 0.16)		0.42 (–0.15 to 0.15)	
			Without comorbidities	0.48 (0.36 to 0.58)		0.48 (–0.21 to 0.21)		0.49 (–0.21 to 0.21)		0.51 (–0.20 to 0.20)	
	**Spain**										
		**Sex**									
			Male	0.63 (0.53 to 0.71)		0.64 (–0.34 to 0.34)		0.60 (–0.34 to 0.34)		0.57 (–0.34 to 0.34)	
			Female	0.78 (0.71 to 0.83)		0.83 (–0.39 to 0.39)		0.86 (–0.39 to 0.39)		0.85 (–0.39 to 0.39)	
		**Age group (years)**									
			>65	0.69 (0.60 to 0.76)		0.73 (–0.40 to 0.40)		0.80 (–0.40 to 0.40)		0.81 (–0.40 to 0.40)	
			18-64	0.81 (0.74 to 0.86)		0.83 (–0.33 to 0.33)		0.85 (–0.36 to 0.36)		0.83 (–0.36 to 0.36)	
	**Brazil**										
		**Sex**									
			Male	0.84 (0.81 to 0.87)		0.81 (–0.34 to 0.34)		0.75 (–0.34 to 0.34)		0.66 (–0.34 to 0.34)	
			Female	0.86 (0.82 to 0.89)		0.82 (–0.32 to 0.32)		0.78 (–0.32 to 0.32)		0.70 (–0.31 to 0.31)	
		**Age group (years)**									
			>65	0.74 (0.69 to 0.79)		0.69 (–0.29 to 0.2)		0.69 (–0.29 to 0.29)		0.70 (–0.29 to 0.29)	
			18-64	0.70 (0.63 to 0.76)		0.65 (–0.25 to 0.25)		0.65 (–0.25 to 0.25)		0.60 (–0.25 to 0.25)	
		**Ethnicity**									
			White	0.86 (0.83 to 0.89)		0.84 (–0.33 to 0.33)		0.78 (–0.33 to 0.33)		0.71 (–0.34 to 0.34)	
			Black or Brown	0.84 (0.80 to 0.87)		0.80 (–0.31 to 0.31)		0.75 (–0.31 to 0.31)		0.68 (–0.31 to 0.31)	

**Table 2:** Results of 1-year (June 2015 to June 2016) forecasts for the number of asthma hospitalizations (according to age, sex, ethnicity, and presence of comorbidities) based on autoregressive integrated moving average models including common cold–related Google Trends data and asthma hospitalizations from the previous 3 years.

Categories					Results for number of predicted and observed hospitalizations, correlation (95% CI)		Average difference in the absolute numbers of predicted and observed weekly hospitalizations, average		Weeks with observed hospitalizations outside the predicted 95% CIs, n (%)
**Pseudo-influenza syndrome topic**									
	**Portugal**								
		**Sex**							
			Male	0.52 (0.26-0.71)		6.5		1 (1.9)	
			Female	0.75 (0.57-0.85)		26.3		5 (9.6)	
		**Age group (years)**							
			>65	0.70 (0.51-0.83)		14.9		6 (11.5)	
			18-64	0.56 (0.38-0.70)		27.8		7 (13.5)	
		**Comorbidities**							
			With comorbidities	0.66 (0.45-0.80)		21.9		1 (1.9)	
			Without comorbidities	0.68 (0.52-0.81)		9.5		1 (1.9)	
	**Spain**								
		**Sex**							
			Male	0.51 (0.22-0.72)		49.1		2 (3.9)	
			Female	0.83 (0.65-0.92)		111.2		12 (23.1)	
		**Age group (years)**							
			>65	0.88 (0.74-0.95)		101.9		12 (23.1)	
			18-64	0.76 (0.59-0.88)		30.1		7 (13.5)	
		**Sensitivity analyses by age group (years)**							
			>65	0.85 (0.73-0.91)		71.3		3 (5.7)	
			45-64	0.89 (0.78-0.94)		10.8		0 (0)	
			18-44	0.85 (0.72-0.92)		11.8		18 (1.8)	
	**Brazil**								
		**Sex**							
			Male	0.91 (0.83-0.94)		75.1		0 (0)	
			Female	0.89 (0.81-0.93)		71.8		0 (0)	
		**Age group (years)**							
			>65	0.83 (0.71-0.90)		32.8		1 (1.9)	
			18-64	0.82 (0.69-0.89)		44.6		1 (1.9)	
		**Sensitivity analyses by age group (years)**							
			>65	0.87 (0.78-0.92)		20.7		3 (5.7)	
			45-64	0.78 (0.63-0.88)		19.3		2 (3.8)	
			18-44	0.74 (0.57-0.85)		22.1		0 (0)	
		**Ethnicity**							
			White	0.92 (0.84-0.95)		40.8		0 (0)	
			Black or Brown	0.87 (0.75-0.93)		71.1		0 (0)	

## Discussion

### Principal Findings

In this study, we assessed the correlations of GT data and the performance of GT-based models in different subgroups of patients (as defined by clinical and demographic characteristics: age, sex, ethnicity, and presence of comorbidities) using the case study of asthma. Overall, our results point out that GT-based models may not necessarily have the same performance in all subgroups of patients, highlighting that GT data may vary across different segments of users. In fact, we observed stronger correlations between GT data and asthma hospitalization data or between forecasted and observed hospitalizations when assessing admissions of women or patients without comorbidities. Less consistent results were observed, in particular in Brazil, according to age group.

Overall, studies using GT data for surveillance purposes have obtained mixed results. On the one hand, GT has been shown to have an effective potential to monitor the spread of infectious diseases, track public interest in health-related topics, and identify emerging trends in public health [37,38]. However, there have also been instances where GT has shown inconsistencies or failed to provide accurate predictions, emphasizing the need to carefully interpret the data [23]. In part, these failures may be related to differences in the composition of internet users compared with that of patients with a particular disease. Although we cannot necessarily generalize the results observed in the use case of asthma hospitalizations to other conditions or countries, this paper is relevant from a methodological point of view, as it demonstrates, through a case study, how the association between GT and disease data is not always the same for all groups of individuals, pointing to the need to study these associations according to the characteristics of the patients.

This study is also relevant for asthma care, as this was the condition we particularly assessed. Regarding our findings of the performance of GT-based models in the distinct subgroups of asthma hospitalizations, we observed relevant gender-related differences. In fact, women have higher asthma prevalence, severity, and health care utilization than men [39,40]. The better correlations and model performance observed in female admissions may be related to the higher prevalence of asthma in this population. On the other hand, women often exhibit more proactive health information–seeking behaviors, with a particular emphasis on their own health and well-being as well as that of their families—which may partly explain the higher internet use by women than men [41]. In addition, in some cultures, women may have primary caregiving responsibilities for family members’ health, including managing asthma [42]. This can also contribute to increasing interest and information-seeking behavior and enhance engagement with online platforms, possibly explaining the higher correlations and better performance of models in admissions of women.

Younger adults, especially those who are generally healthy, may exhibit different health-seeking behaviors than older adults or individuals with chronic illnesses [43]. Younger adults may tend to be more proactive in seeking health care information online and to be more likely to use search engines [44], in part given their historically higher access and rates of internet use [44] (which can be attributed to factors such as greater digital literacy, increased reliance on technology for information and communication, and higher rates of smartphone ownership [44,45]). However, that access has also been proliferating very quickly among older adults, who may possibly be more concerned about their health [46]. These changing patterns may partly explain the heterogeneity of our results obtained on age groups, with higher correlations found for younger adults in contrast with better performance of forecasting models in admissions of older adults. Such a pattern was also observed when performing separate analyses for age groups of 15 to 44-year-olds and 45 to 64-year-olds in Spain and Brazil. All things considered, our findings may offer insights into digital divides, hinting at disparities in internet access, digital literacy, and health information–seeking behaviors across demographic groups. The use of GT-based tools for complementing surveillance systems may have important implications in terms of health equity, considering the discrepancies in internet access across clinical and demographic subgroups.

Information on the presence of comorbidities or ethnicity of patients was only available for one country each (Portugal and Brazil, respectively). The presence of comorbidities has been associated with worse health outcomes for asthma admissions [47]. In addition, we observed a less pronounced seasonal pattern in Portuguese hospitalizations of individuals with comorbidities than in those without, potentially explaining the worse performance of GT-based models in forecasting those admissions. Small differences were observed regarding ethnicity, with a slightly better performance of models for Whites in Brazil, possibly reflecting different regional demographics, internet use patterns, or health care–seeking patterns.

### Strengths and Limitations

Several limitations should be discussed. First, the differences observed in the performance of GT across different subgroups are not necessarily generalizable to other countries and conditions (eg, we cannot state that GT-based models always display better performance when considering data from female participants). Second, data availability on hospitalizations was limited to 3 countries and, regarding the presence of comorbidities or ethnicity, we only had that information for Portugal and for Brazil, respectively. In addition, the small frequency of weekly admissions precluded the comparison of the performance of the models’ unspecific sets of Charlson comorbidity groups. Third, GT provides data on search term popularity and relative interest (ie, GT presents searches as a relative volume instead of as an absolute number of searches), which makes comparisons between queries difficult and reveals less information about the absolute search interest in each aspect being assessed. In addition, it does not provide detailed information about the context or intent behind the searches, thus making it prone to bias due to possible media coverage [48]. In the particular context of this study, we were not able to quantify the number of searches on the “common cold” resulting from individuals experiencing cold symptoms versus reflecting other intentions (eg, search for news on the common cold). This lack of specificity can make it challenging to establish a causal link between search behaviors and the studied outcomes. However, this is an inherent limitation of GT, and our goal was not so much to establish an association between searches on “common cold” and asthma hospitalizations but rather to assess how that association varies considering different subgroups. Finally, during the assessed period, there was an increase in the use of the internet. However, we applied time series analysis methods, removing the estimated trend components for both GT and hospitalization data.

This study also had several strengths. In particular, this study has an important novelty component—to the best of our knowledge, this is the first time that the performance of GT-based models has been investigated across diverse demographic and clinical subgroups, with relevant potential implications for considering digital divides and health equity–related aspects in interpreting results of GT-based tools. In addition, we assessed 3 different countries (1 in Europe and 1 in South America) using nationwide data for a period of 5 years. We applied 2 different strategies to retrieve common cold–related GT data—GT data on the *pseudo-influenza syndrome* topic and search terms regarding the *common cold*—which obtained comparable results. We examined asthma as a case study since (1) asthma, in comparison with other diseases (such as COVID-19), is less subject to a high or variable media coverage, thus not particularly biased for GT data [30,48]; (2) the relationship between asthma admissions and GT data on the common cold has been already established [19]; and (3) the influence of patients’ characteristics on asthma outcomes has been assessed [20]. Although this study relied on a case study on asthma admissions, there is potential application of this methodology to other diseases and segments of the population to better understand the context in which GT-based models can be better applied.

### Conclusions

In this study, we observed better performance of models forecasting asthma hospitalizations in women, White individuals (Brazil), and patients without comorbidities (Portugal), suggesting that the models based on GT may perform differently in subgroups of participants, which may indicate variations in the patterns of health-related information seeking among different segments of internet users. Although GT data have increasingly been assessed as a potential complementary tool to more classical surveillance approaches, determining the best practices for using GT data and understanding its limitations requires exploring in which segments of users it performs better. Although this study assessed the use case of asthma in 3 countries and shows differences in different segments of the population, future studies should explore how GT-related models may differentially perform in accordance with other variables, such as sociodemographic variables (like age, gender, education, income, urban/rural context, underserved populations), as well as to test differences observed in other diseases, countries, and clinical data sources. This study contributes to advancing our understanding of the complexities inherent in the infodemiology field and hints at the need to consider population subgroups and health contexts for the applicability of GT-based surveillance systems.

## Appendix

Multimedia Appendix 1 Parameters used for autoregressive integrated moving average models and results of a 1-year (June 2015 to June 2016) forecasts for the number of asthma hospitalizations.

## Abbreviations

ACSS: Administração Central do Sistema de Saúde
ARIMA: autoregressive integrated moving average
DATASUS: Departamento de Informática do Sistema Único de Saúde
GT: Google Trends
ICD-9-CM: International Classification of Diseases, Ninth Revision, Clinical Modification
ICD-10-CM: International Classification of Diseases, Tenth Revision
