# Supplementary material for: Impact of Demographic and Clinical Subgroups in Google Trends Data: Infodemiology Case Study on Asthma Hospitalizations
Source: J Med Internet Res. 2025 Mar 10;27:e51804. doi: 10.2196/51804 (PMC11933767; doi:10.2196/51804)
Supplement: Multimedia Appendix 1 [file jmir_v27i1e51804_app1.docx]

**Table S1 -** Parameters used for autoregressive integrated moving average models

| **Model** | **Parameters** | **Ljung-Box *p-value*** | **AIC** | **BIC** | **MAE [a]** | **RMSE [b]** |  |
| --- | --- | --- | --- | --- | --- | --- | --- |
| ***Portugal*** |  |  |  |  |  | |  |
| **Sex** |  |  |  |  |  | |  |
| **Male** |  |  |  |  |  | |  |
|  | (4,0,2)(0,1,1)[52] | 0.050 | 1172.6 | 1199.99 | 18.96 | | 24.47 |
| **Female** |  |  |  |  |  | |  |
|  | (3,0,2)(0,1,1)[52] | 0.688 | 1438.98 | 1463.33 | 20.31 | | 23.61 |
| **Age group** |  |  |  |  |  | |  |
| **>65 years** |  |  |  |  |  | |  |
|  | (5,0,2)(0,1,1)[52] | 0.798 | 1258.04 | 1288.47 | 10.92 | | 13.56 |
| **18-64 years** |  |  |  |  |  | |  |
|  | (4,0,0)(0,1,1)[52] | 0.159 | 1420.14 | 1441.44 | 17.21 | | 21.05 |
| **Comorbidities** |  |  |  |  |  | |  |
| **With comorbidities** |  |  |  |  |  | |  |
|  | (3,0,2)(0,1,1)[52] | 0.016 | 1381.72 | 1406.07 | 21.52 | | 29.17 |
| **Without comorbidities** |  |  |  |  |  | |  |
|  | (3,0,2)(0,1,1)[52] | 0.686 | 1367.13 | 1391.48 | 14.32 | | 20.03 |
| ***Spain*** |  |  |  |  |  | |  |
| **Sex** |  |  |  |  |  | |  |
| **Male** |  |  |  |  |  | |  |
|  | (4,1,3)(0,2,0)[52] | 0.002 | 1097.82 | 1121.45 | 36.97 | | 48.47 |
| **Female** |  |  |  |  |  | |  |
|  | (2,1,2)(0,2,1)[52] | 0.001 | 1145.55 | 1163.92 | 61.38 | | 80.34 |
| **Age group** |  |  |  |  |  | |  |
| **>65 years** |  |  |  |  |  | |  |
|  | (3,2,4)(0,2,1)[52] | 0.002 | 1081.53 | 1107.68 | 142.72 | | 154.42 |
| **18-64 years** |  |  |  |  |  | |  |
|  | (3,2,4)(0,1,1)[52] | 0.005 | 1408.69 | 1438.99 | 18.49 | | 22.76 |
| ***Brazil*** |  |  |  |  |  | |  |
| **Sex** |  |  |  |  |  | |  |
| **Male** |  |  |  |  |  | |  |
|  | (4,1,1)(1,1,1)[52] | 0.200 | 1823.53 | 1850.86 | 131.01 | | 186.75 |
| **Female** |  |  |  |  |  | |  |
|  | (4,1,0)(0,1,1)[52] | 0.297 | 1790.94 | 1812.2 | 60.92 | | 71.96 |
| **Age group** |  |  |  |  |  | |  |
| **>65 years** |  |  |  |  |  | |  |
|  | (5,1,1)(0,1,1)[52] | 0.746 | 1451.65 | 1478.98 | 20.68 | | 26.27 |
| **18-64 years** |  |  |  |  |  | |  |
|  | (4,1,1)(0,1,1)[52] | 0.272 | 1602.99 | 1627.28 | 40.31 | | 52.23 |
| **Ethnicity** |  |  |  |  |  | |  |
| **White** |  |  |  |  |  | |  |
|  | (4,1,1)(0,1,1)[52] | 0.837 | 1657.56 | 1681.86 | 55.56 | | 78.84 |
| **Black or brown** |  |  |  |  |  | |  |
|  | (5,1,1)(0,1,1)[52] | 0.326 | 1768.99 | 1796.32 | 105.73 | | 139.22 |
|  |  |  |  |  |  | |  |
|  |  |  |  |  |  | |  |

[a] MAE: mean absolute error; [b] RMSE: root mean squared error

**Table S2** - Results of 1-year (June 2015 to June 2016) forecasts for the number of asthma hospitalizations in a sensitivity analysis by age group in Spain and Brazil (the number of weeks with no events precluded this analysis to be performed for Portuguese hospitalizations)

|  | **Correlation (95% CIs) between the number**  **of predicted and observed hospitalizations [a]** | **Average difference in the absolute numbers**  **of predicted and observed weekly**  **hospitalizations, N [b]** | **Weeks with observed hospitalizations**  **outside predicted 95% CIs, n (%) [c]** |
| --- | --- | --- | --- |
| ***Pseudo-influenza syndrome topic*** | | | |
| ***Spain*** |  |  |  |
| **Age group *** |  |  |  |
| **>65 years** |  |  |  |
|  | 0.85 (0.73-0.91) | 71.3 | 3 (5.7) |
| **45-65 years** |  |  |  |
|  | 0.89(0,78-0.94) | 10.8 | 0 (0) |
| **18-44 years** |  |  |  |
|  | 0.85(0.72-0.92) | 11.8 | 1 (1.8) |
| ***Brazil*** |  |  |  |
| **Age group *** |  |  |  |
| **>65 years** |  |  |  |
|  | 0.87 (0.78-0.92) | 20.7 | 1 (1.9) |
| **45-65 years** |  |  |  |
|  | 0.78(0.63-0.88) | 19.3 | 2 (3.8) |
| **18-44 years** |  |  |  |
|  | 0.74(0.57-0.85) | 22.1 | 0 (0) |
